# Detection of Interleukin-6 Protein Using Graphene Field-Effect Transistor

**DOI:** 10.3390/bios13090834

**Published:** 2023-08-22

**Authors:** Manoharan Arun Kumar, Ramasamy Jayavel, Shanmugam Mahalingam, Junghwan Kim, Raji Atchudan

**Affiliations:** 1 Department of Electrical, Electronics and Communication Engineering, School of Technology, Gandhi Institute of Technology and Management (GITAM), Bengaluru 561203, Karnataka, India; 2 Centre for Crystal Growth, Anna University, Chennai 600025, Tamil Nadu, India; rjvel@annauniv.edu; 3 Department of Materials System Engineering, Pukyong National University, Busan 48513, Republic of Korea; shanmugam82@pknu.ac.kr (S.M.); junghwan.kim@pknu.ac.kr (J.K.); 4 School of Chemical Engineering, Yeungnam University, Gyeongsan 38541, Republic of Korea; 5 Department of Chemistry, Saveetha School of Engineering, Saveetha Institute of Medical and Technical Sciences, Chennai 602105, Tamil Nadu, India

**Keywords:** graphene field-effect transistor, Dirac point, electrical characteristics, Interleukin-6 protein

## Abstract

Universal platforms to analyze biomolecules using sensor devices can address critical diagnostic challenges. Sensor devices like electrical-based field-effect transistors play an essential role in sensing biomolecules by charge probing. Graphene-based devices are more suitable for these applications. It has been previously reported that Graphene Field-Effect Transistor (GFET) devices detect DNA hybridization, pH sensors, and protein molecules. Graphene became a promising material for electrical-based field-effect transistor devices in sensing biomarkers, including biomolecules and proteins. In the last decade, FET devices have detected biomolecules such as DNA molecules, pH, glucose, and protein. These studies have suggested that the reference electrode is placed externally and measures the transfer characteristics. However, the external probing method damages the samples, requiring safety measurements and a substantial amount of time. To control this problem, the graphene field-effect transistor (GFET) device is fabricated with an inbuilt gate that acts as a reference electrode to measure the biomolecules. Herein, the monolayer graphene is exfoliated, and the GFET is designed with an in-built gate to detect the Interleukin-6 (IL-6) protein. IL-6 is a multifunctional cytokine which plays a significant role in immune regulation and metabolism. Additionally, IL-6 subsidizes a variability of disease states, including many types of cancer development, and metastasis, progression, and increased levels of IL-6 are associated with a higher risk of cancer and can also serve as a prognostic marker for cancer. Here, the protein is desiccated on the GFET device and measured, and Dirac point shifting in the transfer characteristics systematically evaluates the device’s performance. Our work yielded a conductive and electrical response with the IL-6 protein. This graphene-based transducer with an inbuilt gate gives a promising platform to enable low-cost, compact, facile, real-time, and sensitive amperometric sensors to detect IL-6. Targeting this pathway may help develop treatments for several other symptoms, such as neuromyelitis optica, uveitis, and, more recently, COVID-19 pneumonia.

## 1. Introduction

Recently, the detection of the electrical conduction of biological species and chemical sensors by advanced nanomaterial-based sensor devices, like carbon nanotubes, graphene, and silicon nanowires, has garnered substantial attention [1,2] for clinical diagnostic and genomics applications. The conventional optical detection technique in the past decade needed more advanced knowledge and complex classification processes. Susceptible electrical detection methods are used to sense the biological species and chemical sensors because the surface-to-analyte or analyte-to-analyte fastenings are very near to the channel. Carbon nanotube field-effect transistors (CNTFETs) and graphene field-effect transistors (GFETs) are the most promising applicants for label-free detection [3,4,5,6]. These transistors have small diameters and high aspect ratios in detecting micro-molecules because the drain current values depend on their diameter and electrode metal working function [7].

Carbon-based device characteristics face some problems in terms of reliability, and a carbon nanotube’s diameter controls are a significant problem [8]. Efforts have been made to resolve these difficulties associated with carbon nanotubes, in order to synthesize the materials on quartz substrates and use them for logic circuits [9]. Furthermore, CNTFETs are exposed in air and electrolytes to demonstrate the p-type characteristics [10]. Due to their band structure, CNT-based devices have faced limitations that it can be able to sense only positively charged biomolecules in electrolytes [11]. Graphene is a single-layer carbon material and has tremendous potential to resolve the difficulties with CNTFETs. Graphene has high electron mobility and large carrier capacities at room temperature without doping, which leads to good transconductance, resulting in the manufacturing of susceptible sensor devices. The surface conditions are inclined towards the electrical characteristics of graphene with high sensitivity; due to this fact, the graphene field-effect transistors (GFETs) were used for detecting gas molecules [12,13,14,15]. Moreover, the investigation has been carried out using GFETs for detecting chemical and biological species because of their unique electrical properties [16]. Furthermore, these devices were broadly utilized for the detection of DNA hybridization [17,18,19], pH sensors [20,21], glucose detection [22], and protein [23,24,25,26,27].

Recently, sensing measurements have received considerable attention using graphene-based devices because of the direct exposure to the electrolyte. While sensing the biological species, it has to be noted that the electric window of the material is broad, which means that it should be hard to oxidize in solution. Electrolyte-gated GFETs have exposed good electrical characteristics [28]. They also have thin top-gate insulators with good dielectric constants, which are more helpful in measuring ionic solutions. The GFET devices were absorbed in the electrolyte solution, and they were positioned using silicone rubber to allow the graphene’s channel surface to be filled with analytes and buffer solutions for electrical measurement and sensing. The ambipolar behavior of the GFET device was analyzed from both back-gate and top-gate operations. The results found that the top gate with a thick HfO_2_ layer was a perfect match to investigate the biomolecules [29]. In electrolyte-gated GFETs, the external electrode was used as a reference electrode to measure the charge transfer of the device during the biomolecules present on the surface of the graphene channel. The reference electrode plays a significant role in detecting the biomolecules introduced in the solution. The charges of the molecule modulated the current in the GFETs, and the reference electrode measured it. Based on the charged molecules (positive or negative) adhered to the surface of the channel, the Dirac point was shifted. Compared with the essential biosensors, the electrical measurements using GFET devices will reduce the time and cost.

The basic principle of GFET sensors to identify the chemical or biological species is to adsorb the molecules on the graphene surface, which acts as a donor or acceptor electron, resulting in a change in conductance [30,31,32]. To investigate the electrical characteristics of GFET biological sensors, it is to be noted that the device should be operated under a low-electrical field, which ensures the graphene avoids biomolecule oxidization. In this study, an attempt was made to apply GFETs to detect the chemical and biological samples. The GFETs behavior with inbuilt gates has been studied to show that they have outstanding transfer characteristics. Moreover, the protein-dependent conductance characteristics have been analyzed and could be able to detect surface-protein adsorption electrically. Thus, the electrical detection of biomolecules has been demonstrated with GFET devices. The monolayer graphene was obtained using a mechanical exfoliation method from the natural graphite. The device was fabricated on a SiO_2_ layer (90 nm thick) and thermally grown on a silicon substrate. The inbuilt gate was fabricated as a reference electrode to avoid species damage.

In this work, we have fabricated a GFET with an inbuilt gate and analyzed the conductance changes by Interleukin (IL-6) protein sample adsorption. Herein, the reference electrode was fabricated as an inbuilt gate to avoid sample damages, external disturbances, and to increase the effectiveness of the sensor. For the first time, we desiccated the IL-6 protein samples on the inbuilt gate GFET device. IL-6 is a kind of pleiotropic cytokine that is more important for immune regulation and exercise metabolism. For the inflammatory responses, cytokines play a significant role in the course of immunology, and they create an interaction between the cells. The major function of the cytokine is to transform the intercellular signals to regulate systemic inflammatory responses. It functions as an immunoregulator in the processes of wound healing and immunity. The transfer characteristics of the device have been studied after desiccating; the IL-6 protein has been desiccated on the substrate. The sensor responds to the charge transfer for the interaction of protein samples bound to the GFET substrate and it is reflected in the Dirac point shifting. It is observed that GFETs are promising sensor devices for developing real-time chemical and biological sensors.

## 2. Materials and Methods

### 2.1. Fabrication of Graphene Field-Effect Transistor

The monolayer graphene was exfoliated with Kish graphite using mechanical exfoliation with the help of scotch tape and it was deposited onto a SiO_2_/Si substrate (90 nm). The graphene sheets on the substrate were identified using an optical microscope. Optical microscopy (S8 APO and EC3, Leica Microsystems, Wetzlar, Germany) was used to determine the monolayer graphene on the SiO_2_/Si substrate, and it was confirmed by Raman spectroscopy (532 nm excitation, RAMANplus, Nanophoton, Osaka, Japan). The Raman spectrum of monolayer graphene was explained in the results and discussion. The band structure of monolayer graphene was analyzed and confirmed, then CAD software (Vector Works 2012 tool, A&A Co., Ltd., Tokyo, Japan) was used to design the electrodes interaction and contact pads utilized for the metal contact. We designed the source, drain, gate, and contact pads using a CAD tool based on the monolayer structure and then initiated the fabrication steps.

**Figure 1:**
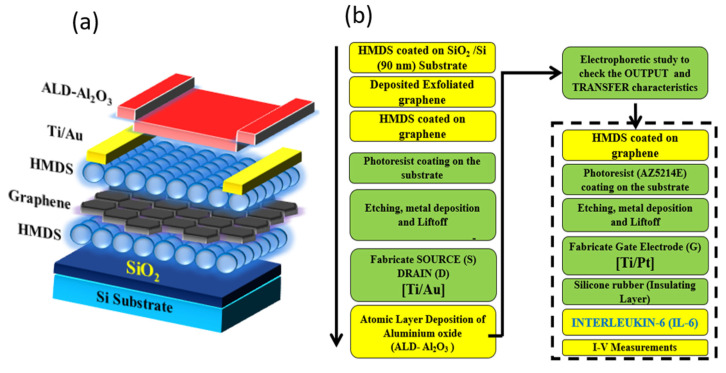
Overview of the fabrication of graphene field-effect transistor device. (**a**) Layers of coating; (**b**) Fabrication steps.

Initially, a hydrophobic hexamethyldisilazane (HMDS, Merck Performance Materials, Darmstadt, Germany) was uniformly coated on the SiO_2_/Si substrate layer. The HMDS layer is a self-assembled protective layer, and it prevents contamination. Furthermore, it improves the electrical characteristics of the device. The complete fabrication steps were clearly demonstrated in this section, and it is schematically shown in Figure 1a,b. The fabrication begins with LOR5A (MicroChem Corp., Newton, MA, USA) deposited on the substrate using the spin coating method, followed by baking the substrate at 180 °C for 4–5 min and acting as a protective layer. The AZ5214E (Merck Performance Materials) was deposited on the substrate and baking the substrate for the next 2–3 min at 110 °C, then the layer acted as a photoresist layer. Maskless scanning lithography (DL-1000/NC2P, NanoSystem Solutions Inc., Okinawa, Japan), with a semiconductor laser with 1 W cm^−2^ power at 405 nm and exposure with dose of 85 mJ cm^−2^, was developed for the proper AZ-resist pattern on the substrate. To create the suitable resist, the substrate was dipped into the standard developer (2.38% solution of tetramethylammonium hydroxide, TMAH, MicroChem Corp.) for about 2–3 min and rinsing the substrate for 30 s in deionized (DI) water. This step helps the substrate to obtain the perfect contact cut to develop the Source and Drain. The electron-gun evaporator (RDEB-1206K, RDEC Inc. Ibaraki, Japan) was used for the evaporation of metal to develop the Source and Drain electrodes (Ti/Au~10/200 nm) on the substrate. The Liftoff method had to be used to remove the metal deposition from the unfavorable regions of the substrate at 80 °C for 1 h using N-methyl-2-pyrrolidone (NMP, MicroChem Corp.) and the substrate was repeatedly washed with acetone and isopropanol. The insulating layer (Al_2_O_3_) was developed by atomic layer deposition (ALD) at 0.8 Å/cycle to passivate the electrodes on the substrate. The Source and Drain electrodes were fabricated on the substrate effectively.

Furthermore, the gate electrode (Ti/Pt~150/50 nm) was fabricated on the substrate in an inbuilt approach. Similar fabrication methods were shadowed to develop the inbuilt gate electrode. Hence, the electrodes are developed on the substrate successfully. The working condition of the device was analyzed by the output and transfer characteristics using a semiconductor parameter analyzer (Keithley, 4200-SCS; LN2 prober-System brain, SB-LN2ps). The detailed study of the device is explained in the Section 3. Furthermore, the tested device was used for the detection of IL-6. Hence, in order to desiccate the protein on the substrate safely, an insulating shield layer was created as a reservoir around the particular region using silicone rubber (TSE382-C, Tanac Co., Ltd., Gifu, Japan). Herein, the insulating layer was constructed to avoid the leakage current.

### 2.2. Detection of Interleukin-6

**Figure 2:**
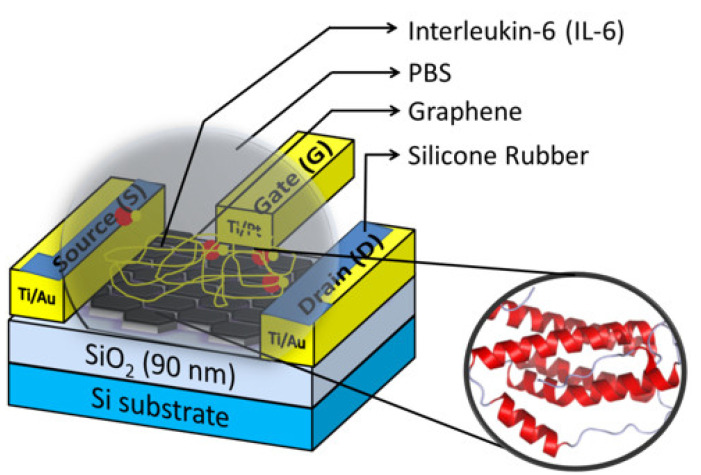
Schematic illustration of Graphene Field-Effect Transistor (GFET)device with Interleukin-6 protein.

The IL-6 was acquired from Greiner Bio-One, (Kremsmunster, Austria). The IL-6 protein samples (10 mM) were prepared appropriately for the phosphate-buffered saline (PBS). The graphene device was initially gestated for 2 h in phosphate-buffered saline (PBS, pH 7.4). The transfer characteristics of the device were measured after a delicate wash with deionized water. The prepared protein samples were desiccated for 1 h 30 min on the GFET at room temperature. The device was completely washed with deionized water and the transfer characteristics were measured. The amperometric measurement of the device was carried out using a semiconductor parameter analyzer. Herein, the back-gate measurement was carried out with SiO_2_/Si substrate, and the top-gate measurement was carried out with the Ti/Pt electrode. The electrical characteristics were analyzed for both the buffer solution and protein sample with proper calibration. Generally, the GFET device was operated at low voltage to prevent oxidization of the electrodes and the graphene channel. The schematic diagram of the complete GFET device desiccated with IL-6 is shown in Figure 2. The transfer characteristics of the device with PBS and IL-6 were measured and discussed in the results and discussion Section 3.2 and Section 3.3.

## 3. Results and Discussion

### 3.1. Identification of Monolayer Graphene

**Figure 3:**
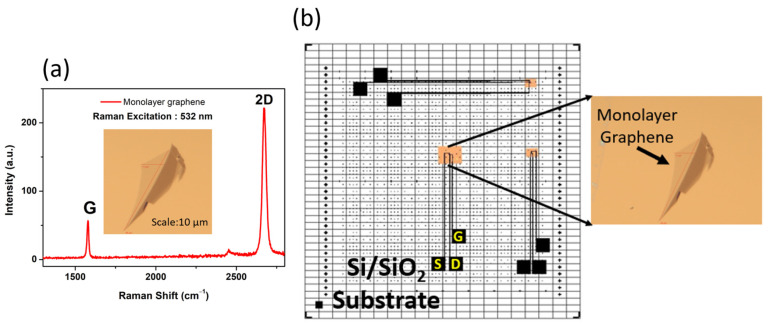
(**a**) Raman spectrum of the monolayer graphene; (**b**) CAD tool designing of the source (S), drain (D), and gate (G) electrodes.

Exfoliated monolayer graphene was placed on a SiO_2_/Si substrate and identified using optical microscopy. The Raman spectrum analysis examined the monolayer structure. The Raman spectrum results of the monolayer structure are based on the position, intensity ratio, and width of the G and 2D peaks observed at 1600 and 2680 cm^−1^, respectively, as shown in Figure 3a. In short, the D-band was observed in the graphene and reduced graphene. Here, the defect-induced D-band was not detected in the mechanically exfoliated graphene. It is indicated that the high structural quality of the sample was obtained from the exfoliation method. Once the monolayer graphene was confirmed on the substrate, the fabrication process was initiated, as mentioned in Section 2.

**Figure 4:**
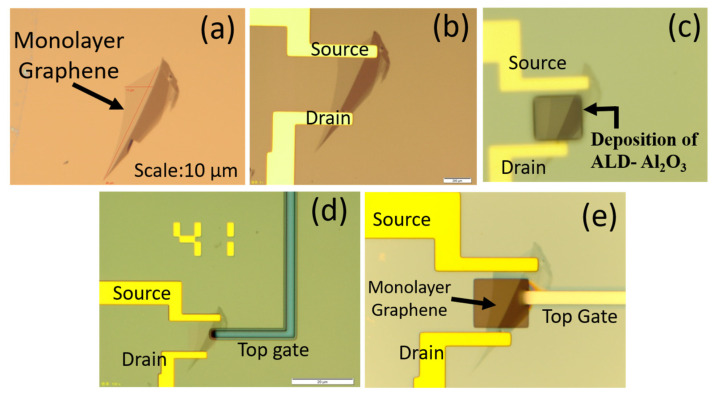
Optical images of the substrate. (**a**) Exfoliated monolayer graphene; (**b**) Source and drain contact; (**c**) Deposition of ALD-Al_2_O_3_; (**d**) Top-gate design; (**e**) Top-gate contact.

The monolayer graphene was identified by designing the electrodes as per the monolayer structure using a CAD tool, as shown in Figure 3b. The source, drain, gate, and contact pads were designed based on the monolayer graphene by CAD tool and then the fabrication steps were initiated. As per the fabrication process mentioned in Section 2, the source and drain were developed as shown in Figure 4a,b. Furthermore, the insulating layer (Al_2_O_3_) was developed using atomic layer deposition (ALD) to passivate the electrodes on the substrate, as shown in Figure 4c. A similar fabrication step was extended to develop the inbuilt gate electrode as a reference electrode, as shown in Figure 4d. Moreover, the fabricated devices should be analyzed using the semiconductor analyzer and then used for sensor applications. Initially, the output characteristics were measured using the analyzer. The output characteristics reveal the excellent ohmic contact between the metal (Ti) interface and monolayer graphene, as reported by Schneider et al. [33]. The output characteristics of the device the sets drain current as a function of the bias voltage at various gate voltages, as shown in Figure 4e.

### 3.2. Output and Transfer Characteristics of the Device

**Figure 5:**
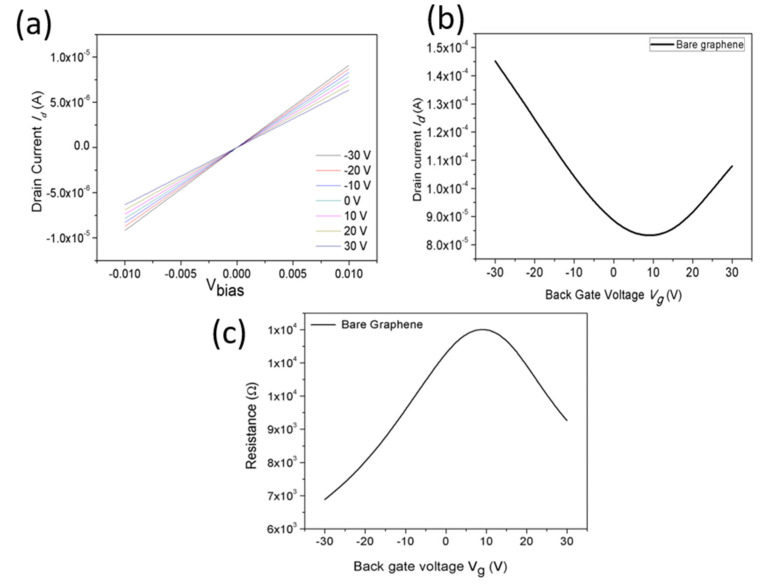
Transfer characteristics of the top-gated graphene device. (**a**) Output characteristics involving drain current as a function of bias voltage measured at various gate voltages ranging from −30 to 30 V in steps of 5 V. (**b**) The transfer curve for bare graphene between the drain current and back-gate voltage at a V_d_ of 0.01 V. (**c**) The resistance curve of the GFET device measured at a V_d_ of 0.01 V.

Graphene is a very sensitive and thin material; while handling the material for making a transistor, it may create Schottky contact between the interfacing metal and graphene. The advantage of the zero energy band gap in graphene and proper handling creates the ohmic contact. The two-dimensional graphene is very sensitive and more effective with metal contact. The interfacing metal Ti/Au was used for the graphene contact. The output characteristics were calculated using sweeping voltage, as shown in Figure 5a, in the range from −30 V to +30 V for the step voltage of 10 V. The output characteristics exposed a linear behavior between the Ti/Au and graphene, and this represents that good contact is established between the graphene and the metals. The transfer characteristics were measured for bare graphene for the drain current and back-gate voltage (at V_d_ = 0.01 V), as shown in Figure 5b. The graph demonstrates that the I_d_ of bare graphene was decreased initially and then it was increased, exhibiting graphene’s electronic behavior, which matches the report of Ohno et al. [34]. The corresponding transfer (resistance vs. back-gate voltage) curve was also plotted, as shown in Figure 5c, authorizing the typical ambipolar behavior of the device. The experimental results of the GFET device demonstrate the charge transfer and doping effect of the graphene at the metal interface. The charge transfer behavior between the graphene and metal contact determines the conduction between the source and drain. The positive gate voltages support the electron concentration in the n-type channel, while the negative voltages support the higher hole concentrations in the p-type channel, which is neutralized at the Dirac point. The charge carriers can be continuously converted from an electron to the holes (or from a hole to an electron) by varying the ambipolar gate voltage, as described by Zhan et al. [35].

### 3.3. Solution-Gated GFET for Interleukin-6 Protein Detection

**Figure 6:**
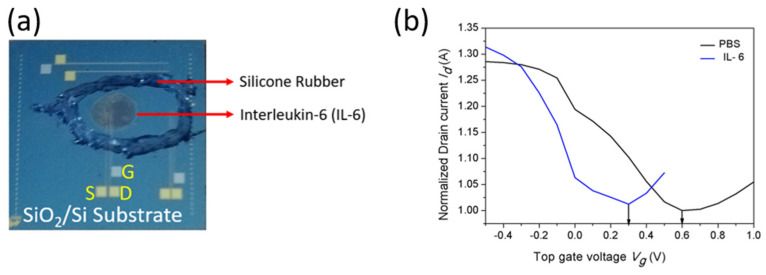
Interleukin-6 Protein desiccated on the GFET Device. (**a**) Photographic image of the GFET device with IL-6 (S—Source; D—Drain; G—Gate). (**b**) Transfer characteristics of the top-gated GFET device for buffer solutions and IL-6 protein.

Generally, for sensor applications, top-gate was comparatively good for device consistency. The top-gated GFETs were fabricated with dielectrics to improve the device’s reliability. The PBS buffer solution was dropped on the substrate and desiccated for 2 h at room temperature. The drain current (I_d_) of the device measured in the buffer solution was plotted against the top-gate voltage V_TG_ (for V_d_ = 0.01 V) and the source-drain voltage, as shown in Figure 6a. Furthermore, the IL-6 protein (10 mM) was prepared in the PBS solution. The prepared protein (IL-6) samples were dropped on the substrate and desiccated for 2 h at room temperature. During this incubation period, the samples were well-desiccated on the substrate. The desiccated samples were washed with DI water to remove the unbounded protein from the substrate. The real-time photographic image of the GFET device with desiccated IL-6 protein is shown in Figure 6b. Now, the transfer characteristics were measured for IL-6 protein with PBS for V_d_ = 0.01 V. It was observed that the transportation of holes and electrons occurred, making the I_d_ values initially decrease and then increase. The conduction of holes and electrons was observed on the device channel for the consequent V_g–_. One possible reason for this behavior is due to the charge impurities present in the graphene underlying the silicon substrate. The instability of the Dirac point shifting reveals the presence of charge impurities. The transfer characteristics were measured separately for buffer solution (PBS) and IL-6 protein with PBS.

The conductance increased for the buffer solution and proved that substantial charge transfer occurred. Moreover, the transfer characteristics were measured for protein samples; Figure 6a shows the conduction changes via the Dirac point shifting towards the negative direction. The results demonstrate the charge transfer, which indicates the absorption of IL-6 protein with PBS onto the graphene surface. The protein samples were directly adsorbed on the graphene surface. The difference between the isoelectric point of the IL-6 pH (=6.96) and buffer solution pH (=7.4) is comparatively small. The minor difference may be directed to some uncharged amino acids of the IL-6 molecules. Furthermore, it is essential to illuminate where the charge transfer occurs and the surface area requirement of protein adsorption. The Dirac point shifting confirmed that the charge type of the adsorbed biomolecules was observed by the GFET device. Under the experimental setup, the holes became carriers in the graphene channel. Hence, protein samples in the phosphate buffer solution were confirmed by the decreased (increased) I_D_ current in the transfer characteristics. In the graphene channel, the hole carrier becomes decreased (increased) whenever it interacts with the positively (negatively) charged proteins. In contrast, the GFETs can equally detect positive charge and negative charge biomolecules, since a Schottky barrier is not produced at the interface between the graphene and electrodes due to its zero-gap nature. These subjects should be investigated for different adsorption protein molecules with other enzymes in order to advance the detection of biomolecules using GFET devices.

## 4. Conclusions

The inbuilt-gated GFET was fabricated and explored for sensing the IL-6 protein in this work. The sensor device was fabricated with a 2D exfoliated monolayer graphene material on the SiO_2_/Si substrate (90 nm) using a CAD tool for the electrode design. We demonstrated a GFET device with output characteristics and transfer characteristics. The output characterization results show that the device contacts are good and working correctly. In the transfer characteristics of the device, the Dirac point was measured and followed by the resistance measurement, showing reproducible results. It is thus suitable for further investigation of IL-6 protein samples. The GFETs measurements showed good transfer characteristics in IL-6 protein samples; their transconductances strongly represented the presence of biomolecules on the surface of the graphene. Moreover, the substantial charge transfer was observed and reflected in the Dirac point shifting, demonstrating the doping effects. The changes that occurred on the graphene channel and the phenomena make one believe that the GFETs can be used for protein detection, and are promising candidates for developing real-time applications such as chemical and biological sensors for detecting biomolecules like COVID-19 pneumonia. For future enhancements, creating an integrated, flexible device and an additional wireless data transfer system would make this device suitable for designing wearable sensor devices during the event of a pandemic.

## Data Availability

Not applicable.
